# Conformation Change, Tension Propagation and Drift-Diffusion Properties of Polyelectrolyte in Nanopore Translocation

**DOI:** 10.3390/polym8100378

**Published:** 2016-10-24

**Authors:** Pai-Yi Hsiao

**Affiliations:** 1 Department of Engineering and System Science, National Tsing Hua University, Hsinchu 30013, Taiwan; pyhsiao@ess.nthu.edu.tw; Tel.: +886-3-516-2247; 2 Institute of Nuclear Engineering and Science, National Tsing Hua University, Hsinchu 30013, Taiwan

**Keywords:** polyelectrolyte, translocation, conformation, tension propagation, drift-diffusion, probability density distribution, molecular dynamics simulations

## Abstract

Using Langevin dynamics simulations, conformational, mechanical and dynamical properties of charged polymers threading through a nanopore are investigated. The shape descriptors display different variation behaviors for the *cis*- and *trans*-side sub-chains, which reflects a strong *cis-trans* dynamical asymmetry, especially when the driving field is strong. The calculation of bond stretching shows how the bond tension propagates on the chain backbone, and the chain section straightened by the tension force is determined by the ratio of the direct to the contour distances of the monomer to the pore. With the study of the waiting time function, the threading process is divided into the tension-propagation stage and the tail-retraction stage. At the end, the drift velocity, diffusive property and probability density distribution are explored. Owing to the non-equilibrium nature, translocation is not a simple drift-diffusion process, but exhibits several intermediate behaviors, such as ballistic motion, normal diffusion and super diffusion, before ending with the last, negative-diffusion behavior.

## 1. Introduction

Polyelectrolytes are charged polymers in solutions. The presence of ions drastically changes the properties of the chains [1,2,3,4,5,6,7,8,9,10,11,12,13,14,15,16]. For example, the chain stiffness can be effectively modified by the electrostatic interaction, and a quantity to describe it is called the “persistence length”, which is defined as the correlation length of the bond vectors on the chain backbone [17]. The persistence length can be split into a sum of the intrinsic persistence length and the electrostatic persistence length [18,19,20,21,22], and the latter one depends on the ion types and concentrations in the solution [23,24,25,26]. Therefore, the ionic conditions influence greatly the behavior of polyelectrolytes, which is much more complicated than neutral polymers. In the past two decades, there are growing demands on understanding the physics of polyelectrolytes threading through a nanopore because the technique offers a promising future for the fast detection of the sequences of DNA molecules (which are known as polyelectrolytes) [27,28,29,30,31,32,33,34,35]. Probably because of the limitation of computing power or for the reduction of the complexity of the system, most of the simulations in the literature studied the translocation problem using neutral polymers [36,37,38,39,40,41,42,43]. The physics are not exactly the same as those for the charged polymers. Recently, we have simulated polyelectrolytes threading through a nanopore [44]. In addition to studying the translocation time and the related quantities, we were able to study variations of the ion distribution and ion condensation during a translocation process, since the ions were explicitly modeled.

The objective of this study is to investigate further the conformations, mechanics and dynamics of the forced translocation of charged polymers through a nanopore. In the previous paper [44], the mean translocation time was shown to scale as $\langle \tau \rangle ∼N^{\alpha }E^{-\delta }$, where *N* is the number of monomers of the chain and *E* is the strength of the driving field inside the pore. The exponents *α* and *δ* were found to be not universal, and the values depend on *E*. We demonstrated that the translocation changes from a near-equilibrium process to a far-from-equilibrium one as *E* increases, through the calculation of chain size [44]. Nonetheless, the morphological variation of the chain during a translocation process has not been studied so far. In this work, we deal with the topic by calculating the shape factor *η*, the asphericity *A* and the prolateness *P* of the chain on the cis and trans sides separately. The definitions of these quantities are given in Section 4.1. It has been reported that the three quantities have the mean values of about 6.4, 0.45 and 0.57 for neutral chains in bulk solutions [26,45,46], whereas for polyelectrolytes, the values are around 7.1, 0.52 and 0.72, respectively [26,47]. Therefore, charged chains are more elongated or stiff than neutral chains. When a charged chain threads through a pore, the space restriction by the pore and the impenetrability of the separating wall render the system very different from the bulk solutions; the situation is very complicated. It is hence important to study the conformation change of the chain during a translocation process [48]. Theorists have predicted a trumpet conformation for the cis-side chain in a weakly-driven condition. If the driving force is strong, the chain section near the pore entrance can be straightened; the sub-chain thus adopts a stem-flower or a strongly-stretching conformation [49,50,51,52].

Polyelectrolyte translocation is usually effectuated by setting a voltage difference across the membrane wall, between the cis and the trans regions [27,31,53,54,55,56,57,58]. Since the solutions are ionic (and hence, electrically conductive) in the two regions, the drop of voltage occurs mainly inside the pore [40,59,60]. It establishes an electric field in the pore, and thus, the field acts only on the charged monomers momentarily passing the pore [36,37,39,41,61,62]. The localized driving field induces unbalanced tension force and, hence, the propagation of tension along the chain. The tension front subdivides the sub-chain on the cis-side into two sections. Near the pore is the first section, which consists of the monomers pulled by the tension force, and the other is the section in which the monomers are not yet influenced by the force [41,49,50]. The study of the waiting time, the time spent for each monomer in the pore, depicts a two-stage dynamics for translocation under a strong driving condition [37,41,63,64,65]. In the first stage, the tension front moves on the sub-chain, and the waiting time is an increasing function of the translocation coordinate *s* (the number of monomers entered into the trans region). When the front reaches the cis-side end, the waiting time arrives at its maximum, and the system enters the second stage of tail retraction, characterized by a decrease of the waiting time function. In Section 4.2 and Section 4.3, we calculate the tension force on the threading chains and the waiting time function. The tension front is located during a translocation process and analyzed with the direct and the contour distances of the monomer to the pore entrance.

From a theoretical point of view, polymer translocation can be mapped to a biased diffusion problem: a diffusive particle driven in one-dimensional (1D) space of the translocation coordinate to overcome an entropic barrier [66,67,68,69]. The process is usually described by a Fokker–Planck equation under different boundary conditions [32,70,71]. The problem is understood by solving the probability distribution of *s* as a function of time *t* and finding how the coordinate evolves to its final value, *N*. The diffusion coefficient in the Fokker–Planck equation is generally assumed to be a constant, while the drifting rate is estimated by the terminal velocity derived from the external driving force against the entropic force resulting from the “tug of war” between the equilibrated sub-chains in the cis and the trans regions and, hence, depends on *s* [66,67,68,69,72]. If the threading speed is high, the equilibrium assumption for the sub-chains in the two regions is no longer held. The dynamics turn out to be of far-from-equilibrium and are characterized by a strong memory effect [73,74,75]. Some theorists have employed fractional Fokker–Planck equations to treat the problem [76,77]. Others developed tension-propagation theory, using a tensile blob picture, to analyze the anomalousness [49,50,52,78,79,80]. It was found that the average drift in translocation is characterized by a nonlinear memory kernel through the analysis of a generalized Langevin equation [81]. Moreover, the dynamics of the chain across the pore were shown to be asymmetric: the sub-chain on the cis side is subject to a pulling force, and the dynamics is described by a 1D dynamical equation with fast evolution, whereas the trans-side sub-chain is crowded near the pore exit and diffuses slowly into the open semi-space through a 3D diffusion equation [82,83]. Thus, the drift-diffusion behavior of a threading chain is determined by many effects, which are non-symmetric, non-equilibrated and anomalous. It makes the problem very difficult to analyze and predict. Computer simulation is a powerful tool that studies translocation under well-controlled numerical conditions [36,37,38,39,40,41,42,43,84,85]. It is hence an effective way to study the drift-diffusion properties of translocation. However, as far as we know, the direct tackling of the problem by simulations is still scarce in the literature. In Section 4.4, we study the drifting velocity of the chain in different driving fields. The diffusion behavior is investigated by calculating the variance of the translocation coordinate. The probability density distribution p(s,t) is analyzed and fitted with a log-normal function. The conclusions are given in Section 5. All of the symbols used in this work are listed at the end of the paper.

## 2. Model and Setup

The polyelectrolyte is modeled as a charged bead-spring chain of *N* monomers. Each monomer dissociates a monovalent cation into the solution and, thus, carries a negative unit charge −e. To simulate a saline solution, an amount of monovalent salt is added into the system. In the solution, the salt molecules dissociate into monovalent cations and monovalent anions. A membrane wall divides the system into two subspaces along the *z*-direction, called the cis and the trans regions, which are connected by a pore punched through the wall. A driving electric field is set inside the pore. In order to save the computing resources, the wall is made hollow, and the wall beads are set to be immobile. The wall thickness is 4.5σ, and the pore radius is 2.25σ, where *σ* is the simulation length unit. The simulation box has dimensions of 48.0σ×49.36σ×200.0σ. The periodic boundary condition is employed.

The excluded volume interactions are modeled by the Weeks–Chandler–Andersen (WCA) potential [86]. The Lennard–Jones parameters in the WCA potential are set to be $\sigma_{\mathrm{bb}}=1.0\;\sigma$ and $\varepsilon_{\mathrm{bb}}=1.2\;k_{B}T$ for the interactions between the mobile beads (the monomers and ions), while the ones between the mobile and the immobile (the wall) beads are set to be $\sigma_{\mathrm{bw}}=1.5\;\sigma$ and $\varepsilon_{\mathrm{bw}}=2.5\;k_{B}T$. Here, $k_{B}$ is the Boltzmann constant, and *T* is the temperature; and $k_{B}T$ serves as the energy unit. The electrostatic interactions between the charged beads (the monomers and ions) are calculated by the particle-particle/particle-mesh Ewald method, and the Bjerrum length, which defines the coupling strength of the electrostatic interaction in a dielectric solution, is set to $\lambda_{B}=3.0\;\sigma$. The monomers are bonded by a harmonic potential with the spring constant $k=600.0\;k_{B}T/\sigma^{2}$ and the equilibrium bond length $\ell_{0}=1.0\;\sigma$. The mass of each mobile beads is set to be *m*.

**Figure 1:**
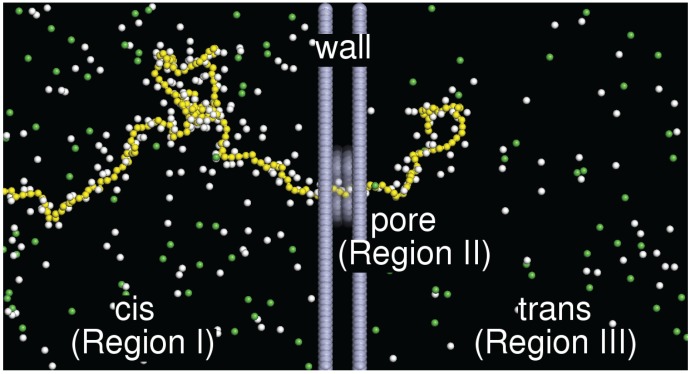
An illustration of the system. The negatively-charged polyelectrolyte is colored in yellow bead-spring chains. The counterions ((+1)-ions) are represented in white beads, and the coions ((−1)-ions) are in green beads. A hallow wall separates the space into the cis region (Region I) and the trans region (Region III), connected by a pore channel (Region II) at the center. An electric field $\vec{E}=-E\hat{z}$ is applied inside the pore where the unit vector $\hat{z}$ points to the right, along the channel axis. The chain threads through the pore and is gradually transported from the cis region to the trans region by the electric field. In order to visualize the chain section inside the pore, the wall beads (in gray color) have been plotted with a certain degree of transparency.

Initially, the chain head section is placed just across the pore and equilibrated with the head end constrained at the pore exit on the trans side. A translocation process is started by removing the constraint and switching on the electric field $\vec{E}=-E\hat{z}$ inside the pore. To guarantee the success of a translocation, a wall potential is set at the pore exit, visible only to the head monomer, which prevents the head monomer moving back to the pore. The electric field then drives the monomers in the pore toward the +z-direction, and the chain is transported gradually from the cis region to the trans one via the pore. In this study, the number of monomers of a chain *N* is varied from 32 to 384. The added salt is fixed at an amount of 256 molecules. The field strength is changed from E=0.2 to $32.0\;k_{B}T/\left(e\sigma \right)$. The simulations are performed using Langevin dynamics simulations [87]. An illustration of the system is given in Figure 1. More details about the model and setup can be found in the paper [44].

## 3. Mapping Simulation Units to Real Units

Mapping a simulation model to a real system is an important task particularly in a coarse-grained simulation. It verifies the goodness of a model and can be done in the following way for our system. In our study, the Bjerrum length is $\lambda_{B}=3.0\;\sigma$. Since $\lambda_{B}$ is 7.14Å in water, simulating an aqueous system can be achieved by setting the length unit *σ* to 2.38Å. The thermal energy $k_{B}T$ is equal to $4.14\times 10^{-21}\;J$ at room temperature (T=300K). It is a suitable unit to describe the energy of an individual particle. The mass unit *m* is set to be 200g/mol because a monomer weighs typically this mass in experiments. For example, the four nucleobases, adenine, thymine, cytosine and guanine, have molar masses of 135.13, 126.12, 111.10 and 151.13g/mol, respectively. Therefore, m=200g/mol represents roughly the mass of a nucleobase or a base pair. To be compatible with the above choice, the time unit $\tau_{u}$ must be assigned to $\sigma \sqrt{m/(k_{B}T)}$, which takes a value of 2.13ps. The charge unit *e* is taken to be $1.602\times 10^{-19}\;C$.

Under this mapping, the wall thickness and the pore diameter are both 1.07 nm (= 4.5 *σ*). The concentration uses the unit $\sigma^{-3}$, which is 123.2mol/L. Thus, the 256 added salt molecules in the simulation box has a concentration equal to:

$$C_{s}=\frac{256}{\left(48\times 49.36\times \left(200.0-4.5\right)\right)\;\sigma^{3}}=0.068\;\mathrm{mol}/L$$

which is of about the same order of the medical saline concentration 0.154mol/L. The unit for the strength of electric field is $k_{B}T/\left(e\sigma \right)$ and is equal to $1.08\times 10^{8}\;V/m$. The range of the simulated field strength thus corresponds to the real value of $2.17\times 10^{7}$ to $3.48\times 10^{9}\;V/m$. It covers the typical strength of a biased electric field in translocation experiments [27,53,54,55,56,57,58]: the bias voltage is typically 100mV, and the pore length is around 0.3 to 10nm, which produces an electric field of the order of about $10^{7}$ to $3.33\times 10^{8}\;V/m$ in the pore. The force quantity has the unit $k_{B}T/\sigma$, which corresponds to a real force of 17.4pN. In Section 4.2, we will see that the bonds on a chain are stretched by tensile forces of order of one to 100 of the force unit, depending on the strength of the driving field. Therefore, the forces correspond to tens to thousands of piconewtons, which are consistent with the experimental estimates [53,55,56].

The persistence length $\ell_{p}$ of our charged chain can be estimated from the Odijk–Skolnick–Fixman theory [18,19], given by:

$$\ell_{p}=\ell_{p,0}+\ell_{p,e}=\ell_{p,0}+\frac{\lambda_{B}}{4{\left(\kappa \ell_{c}\right)}^{2}}$$

where $\ell_{p,0}$ and $\ell_{p,e}$ denote the intrinsic and the electrostatic persistence lengths, respectively, $\ell_{c}$ is the charge distance on the chain backbone and $\kappa =\sqrt{8\pi \lambda_{B}I}$ is the inverse Debye length. In this study, $\ell_{p,0}$ and $\ell_{c}$ are equal to $\ell_{0}$, and the ionic strength *I* is equal to $C_{s}$. It yields $\ell_{p}=18.0\;\sigma$, or 45.2Å in the real length unit. The persistence length is comparable to the one for single-stranded DNA, which is about 20Å in physiological conditions [24]. To simulate double-stranded DNA chains, one needs to add bending potentials to the coarse-grained model to increase the persistence length to an order of 500Å [25].

The mapping of the translocation time to real values is discussed in the Supplementary Materials. The qualitative match of a bunch of important physical quantities, dimensions and scales between the simulations and the experiments justifies the validness of our model.

To shorten the notation, we will describe physical quantities in the (*σ*, *m*, $\tau_{u}$, *e*)-unit system in the following sections if the unit is not specified. For example, E=0.2 is a shortened version of $E=0.2\;\sigma m\tau_{u}^{-2}e^{-1}$, or equivalently, $0.2\;k_{B}T/\left(e\sigma \right)$.

## 4. Results and Discussion

### 4.1. Chain Conformation and Orientation

**Figure 2:**
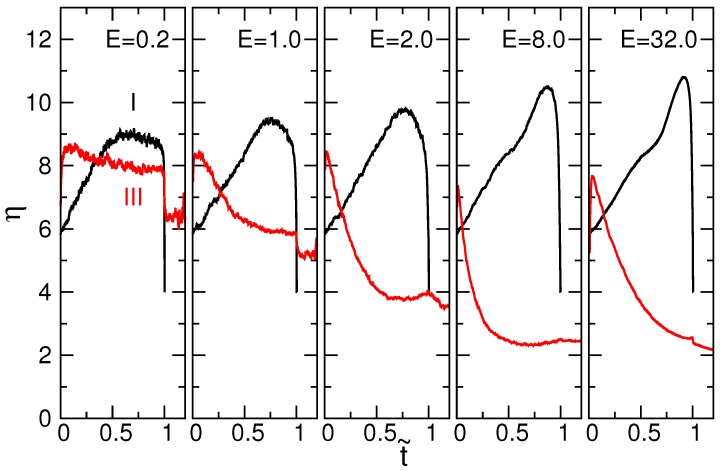
Shape factor $\eta =\langle R_{e}^{2}\rangle /\langle R_{g}^{2}\rangle$ in the cis region (I) and trans region (III) for N=256 as a function of the scaled time $\tilde{t}$ at five driving electric fields *E* whose values are indicated in the plots.

Understanding the conformation change of a chain in a translocation process is important. Here, we use the shape factor to study the variation of the chain conformation. It is defined as:

(1) $$\eta =\frac{\langle R_{e}^{2}\rangle }{\langle R_{g}^{2}\rangle }$$

where $\langle R_{e}^{2}\rangle$ and $\langle R_{g}^{2}\rangle$ are the mean squares of the end-to-end distance and of the radius of gyration, respectively. For a rod-like chain, the shape factor is 12. For an ideal chain, the value is six. If the chain forms a compact globule, such as a sphere or disk-like object, with the chain ends randomly distributed inside it, *η* takes a small value of two (refer to the Supplementary Materials for the derivation). During a translocation, the chain goes across the three regions: the cis region (I), the pore region (II) and the trans region (III). We focus on the conformation change of the chain portions in the cis and trans regions. The obtained shape factors are plotted in Figure 2 as a function of the scaled time $\tilde{t}$ at different electric fields *E*. The chain has 256 monomers, and the scaled time is defined as $\tilde{t}=t/\langle \tau \rangle$, where 〈τ〉 is the mean translocation time.

We can see that $\eta_{I}$ is about six in the cis region (I) at the starting point $\tilde{t}=0$. It indicates that the chain acquires a coiled conformation at the beginning. As the process advances, $\eta_{I}$ increases, and thus, the chain is elongated. The stronger the electric field, the higher the value of $\eta_{I}$. Therefore, the degree of elongation increases with the electric field. Near the end of the process $\tilde{t}=1$, a drastic decrease is observed. It results from a significant decrease in the number of monomers on the cis side. When the number of monomers is small, the shape factor deviates from the above-mentioned typical value. For example, *η* for a three-monomer rod is six. A two-monomer segment has an *η* as small as four.

On the trans side, the shape factor $\eta_{\mathrm{III}}$ starts at a value of around eight and decreases with time. The final value of $\eta_{\mathrm{III}}$ can be as small as two in the strong fields (E≥8.0), which indicates the formation of a compact globule. The globule could be a sphere or a disk. The study [44] has shown that the monomers in the trans region were crowded near the wall under a fast translocation and shaped a pancake-like density profile. Therefore, the result obtained here is derived from the formation of a disk-like object in the strong fields.

We further studied the geometry of the sub-chains by calculating the gyration tensor $\left[G\right]$. The element of the tensor for a given set C of beads is defined by:

(2) $${\left[G\right]}_{\alpha \beta }=\frac{1}{N_{C}}\sum_{i\in C}{\left({\vec{r}}_{i}-{\vec{r}}_{\mathrm{cm}}\right)}_{\alpha }{\left({\vec{r}}_{i}-{\vec{r}}_{\mathrm{cm}}\right)}_{\beta }$$

where *α* and *β* stand for the *x*, *y*, *z* components, $N_{C}$ is the number of beads in the set C, ${\vec{r}}_{i}$ is the position vector of the bead *i* and ${\vec{r}}_{\mathrm{cm}}$ is the center of mass of these beads. The three eigenvalues of $\left[G\right]$ were computed and denoted by $\lambda_{1}$, $\lambda_{2}$ and $\lambda_{3}$. Assume $\lambda_{1}\geq \lambda_{2}\geq \lambda_{3}$. The geometry of the object can be regarded as an ellipsoid, with $\sqrt{\lambda_{1}}$, $\sqrt{\lambda_{2}}$ and $\sqrt{\lambda_{3}}$ representing the length of the three semi-axes, respectively. Two shape descriptors were used. One is the “asphericity”, defined by:

(3) $$A=\frac{{\left(\lambda_{1}-\bar{\lambda }\right)}^{2}+{\left(\lambda_{2}-\bar{\lambda }\right)}^{2}+{\left(\lambda_{3}-\bar{\lambda }\right)}^{2}}{6{\bar{\lambda }}^{2}}$$

where $\bar{\lambda }\equiv \left(\lambda_{1}+\lambda_{2}+\lambda_{3}\right)/3$ is the mean of the eigenvalues [45,46]. It is a dimensionless quantity, which measures the relative extent from a sphere. The value of *A* lies between zero and one. The second descriptor is the “prolateness”, defined by [45,46]:

(4) $$P=\frac{\left(\lambda_{1}-\bar{\lambda }\right)\left(\lambda_{2}-\bar{\lambda }\right)\left(\lambda_{3}-\bar{\lambda }\right)}{{\bar{\lambda }}^{3}}.$$

**Figure 3:**
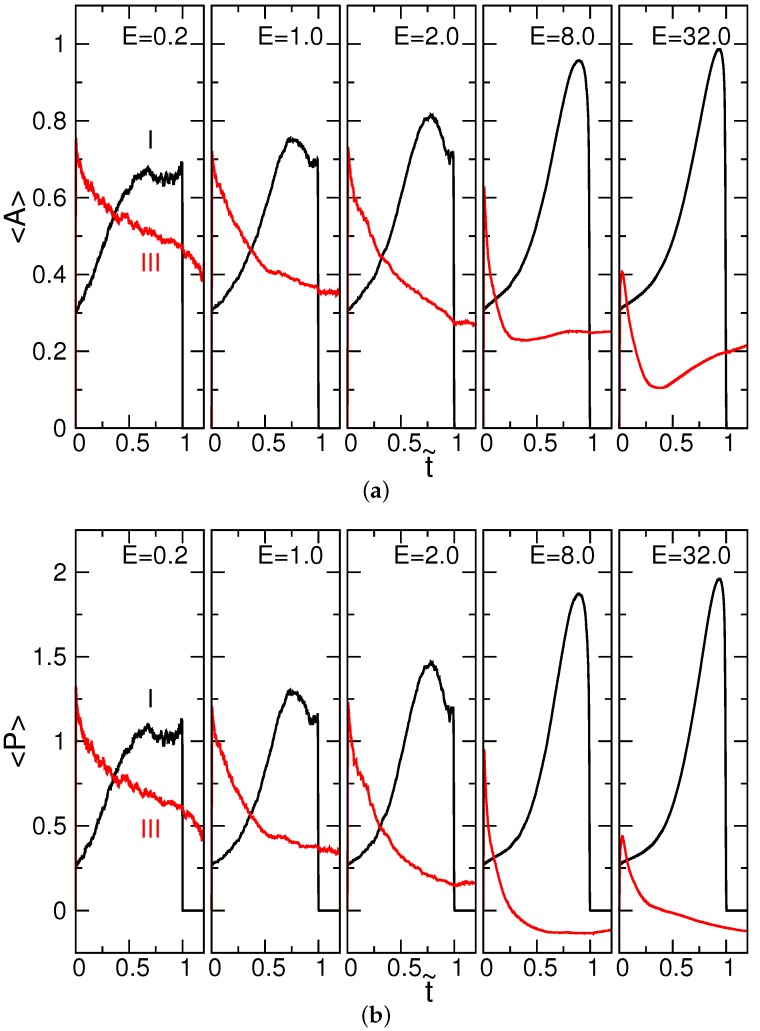
Average variations of (**a**) the asphericity 〈A〉 and of (**b**) the prolateness 〈P〉 in the cis region (I) and trans-region (III) as a function of the scaled time $\tilde{t}$, for N=256 at different driving electric fields *E* (indicated in the plots).

A positive *P* depicts a prolate ellipsoid, whereas a negative *P* depicts an oblate one. The value of *P* falls in the interval [−0.25,2]. A sphere ($\lambda_{1}=\lambda_{2}=\lambda_{3}$) has A=P=0, while a rod (which is an extremely elongated ellipsoid: $\lambda_{1}>0$, $\lambda_{2}=\lambda_{3}=0$) acquires the limiting values (A,P)=(1,2). For a disk-like object ($\lambda_{1}=\lambda_{2}>0$, $\lambda_{3}=0$), the values of *A* and *P* are 0.25 and −0.25, respectively. We studied the average variations of the asphericity 〈A〉 and the prolateness 〈P〉 during a translocation in the cis and trans regions. The results are given in Figure 3.

In the cis region, the starting $\langle A_{I}\rangle$ and $\langle P_{I}\rangle$ are 0.30 and 0.26, respectively. The values are smaller than neutral flexible chains in bulk solutions: 〈A〉=0.431 [45] and 〈P〉=0.541 [46]. Therefore, the geometry of the chain is rounder than a coil. Similar to $\eta_{I}$, the quantities $\langle A_{I}\rangle$ and $\langle P_{I}\rangle$ increase with time, showing a stretch of the sub-chain by the driving electric fields. At the strongest field E=32.0, the two quantities reach the maximum values, one and two, respectively. The sub-chain is thus rod-like near the end of the process.

In the trans region, the prolateness $\langle P_{\mathrm{III}}\rangle$ decreases with $\tilde{t}$. If the field is strong enough (such as E=8.0 and 32.0 in the figure), the value of $\langle P_{\mathrm{III}}\rangle$ can become negative. A geometrical transition hence happened, changing from a prolate object to be an oblate one. The results confirm again the formation of a disk-like object in the trans region. The value of $\langle A_{\mathrm{III}}\rangle$ decreases basically with time, following the change of $\langle P_{\mathrm{III}}\rangle$. However, when the prolate-oblate transition occurs, the geometry crosses over a “spherical” shape, and thus, $\langle A_{\mathrm{III}}\rangle$ shows a minimum. The asphericity then increases with $\tilde{t}$ and, finally, acquires a value of about 0.25, which corresponds to a disk.

The orientations of the sub-chain in the cis and trans regions can be studied by finding the direction of the principal axis. The direction is given by the eigenvector ${\vec{e}}_{1}=\left(e_{1x},e_{1y},e_{1z}\right)$ of the gyration tensor, associated with the largest eigenvalue $\lambda_{1}$. We assumed $e_{1z}>0$ and calculated the polar angle *θ* and the azimuthal angle *ϕ* with respect to the *z*-axis and *x*-axis, respectively, by:

(5) $$\begin{array}{ccc} \theta & = & \tan^{-1}\left(\sqrt{e_{1x}^{2}+e_{1y}^{2}}/e_{1z}\right) \end{array}$$

(6) $$\begin{array}{ccc} \phi & = & \tan^{-1}\left(e_{1y}/e_{1x}\right) \end{array}$$

**Figure 4:**
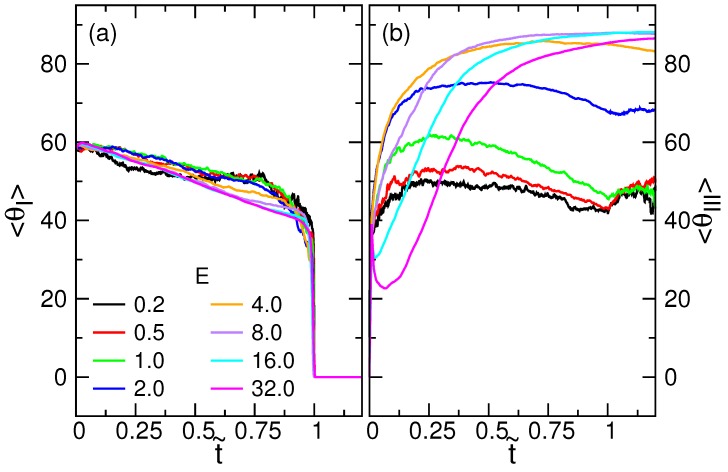
Variations of the averaged polar angle (**a**) $\langle \theta_{I}\rangle$ and (**b**) $\langle \theta_{\mathrm{III}}\rangle$ in degrees ${}^{\circ }$, as a function of the scaled time $\tilde{t}$ at different driving electric fields *E*. The number of monomers of the chain *N* is 256.

The value of *θ* is mapped to the interval between $0^{\circ }$ and $180^{\circ }$ while *ϕ* to the one between $-180^{\circ }$ and $180^{\circ }$. Because of the symmetry in the transverse direction, the averaged 〈ϕ〉-curves fluctuate around $0^{\circ }$. The results are shown in the Supplementary Materials, in Figure S1. The averaged variation of the polar angle, on the other hand, shows structures and is presented here, in Figure 4.

We see that the starting value of $\langle \theta_{I}\rangle$ is around $58^{\circ }$, which is close to $180^{\circ }/\pi$, the expected value for the polar angle of a randomly-pointed vector in the upper hemisphere. For E=0.2, $\langle \theta_{I}\rangle$ displays a plateau at around $52^{\circ }$ when $\tilde{t}$ is in the interval (0.25,0.75). If the field is strong, the polar angle decreases linearly to $40^{\circ }$ in the course of the translocation. The variation of the $\langle \theta_{I}\rangle$ curve depends not much on the filed strength. In contrast, $\langle \theta_{\mathrm{III}}\rangle$ in the trans region depends heavily on *E*. Driven by the strong fields E≥8.0, the angle can approach $90^{\circ }$ when $\tilde{t}$ is near one, showing that the principal axis is about perpendicular to the longitudinal direction. The results are in agreement with the pancake-like profile of the monomer density formed on the trans side of the wall.

The *η*, 〈A〉, 〈P〉 and 〈θ〉 curves on the cis side are dissimilar to the ones on the trans side, particularly when *E* is strong. It can be attributed to the cis-trans dynamical asymmetry where the chain segment is pulled by the tension into the pore, while it is pushed out of the pore to the trans region [82,83]. Theorists have predicted that the conformation of a cis-side sub-chain is trumpet-like under a weakly-driving condition; it changes to be stem-flower-like or of a strongly-stretching figure if the driving force is strong [49,50,51,52]. The calculation of the polar angle shows that the pervaded space for the sub-chain is wider than thought. Therefore, the monomer density distribution looks more like a “shuttlecock” on the cis side, as shown in Figure 11 of [44]. The differences between the density distributions at a weak and at a strong driving force are not as obvious as thought.

### 4.2. Tension Propagation on a Chain

**Figure 5:**
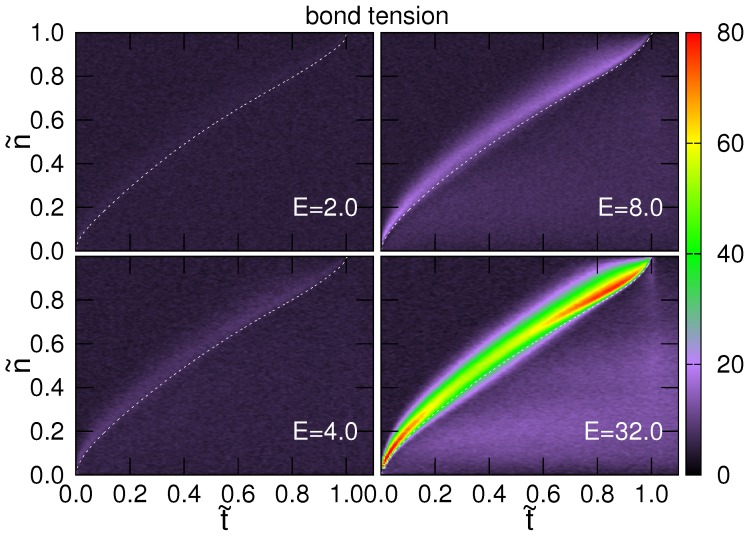
Intensity plots of the bond tension $\langle f_{n}\rangle$ in the scaled $\tilde{t}$-$\tilde{n}$ space for N=256 at four field strengths *E* (indicated in the figures). The strength of tension is represented by color, and the color scale is given at the right of the figure. The dashed line shows the scaled translocation coordinate $\langle \tilde{s}\rangle$, which depicts the progress of threading.

In this section, we study the variation of local tension on a chain backbone during a translocation process. The tension force was calculated via bond stretching by the formula:

(7) $$\langle f_{n}\rangle =k\;\left(\langle \ell_{n}\rangle -\ell_{0}\right)$$

where *n* is the identification (ID) number of the bond, which connects the *n*-th and the (n+1)-th monomers, k=600.0 is the spring constant, $\langle \ell_{n}\rangle$ is the mean length of the *n*-th bond and $\ell_{0}=1.0$ is the equilibrium bond length. Since the tension force is a function of the bond ID and time, the results of the calculation are presented in Figure 5 through an intensity plot where the color represents the strength of the tension. The *x*-axis is the scaled time $\tilde{t}$, and the *y*-axis is the scaled ID number $\tilde{n}=n/N$. The case N=256 is studied. To indicate the progress of translocation, the averaged variation of the scaled translocation coordinate $\langle \tilde{s}\rangle \equiv \langle s\rangle /N$ has been plotted in the figure. The translocation coordinate *s* (denoted by $N_{m,\mathrm{III}}$ in the paper [44]) is defined to be the number of the monomers in the trans-region. Therefore, the region below the $\langle \tilde{s}\rangle$-curve depicts the bonds that have entered the trans-region.

When E≤2.0, the intensity is homogeneous, showing that the bond tensions are uniform on the chain, aside from the fluctuations. As the driving field *E* becomes strong (for example, E=4.0, 8.0 and 32.0 in the figure), a luminous and colorful stripe is displayed above the $\langle \tilde{s}\rangle$-curve and extended from the $\left(\tilde{t},\tilde{n}\right)=\left(0,0\right)$ point to the (1,1) point. It shows a surge of the chain tension. The left boundary of the surge is the tension front. Once the bonds are transferred to the trans region, the tension drops quickly to a background value. The width of the surge increases with time up to $\tilde{t}≃0.8$ at which the tension front reaches the sub-chain end $\tilde{n}=1$; it then shrinks quickly to zero at the end of the translocation. The stronger the driving field, the wider and higher the surge of tension.

**Figure 6:**
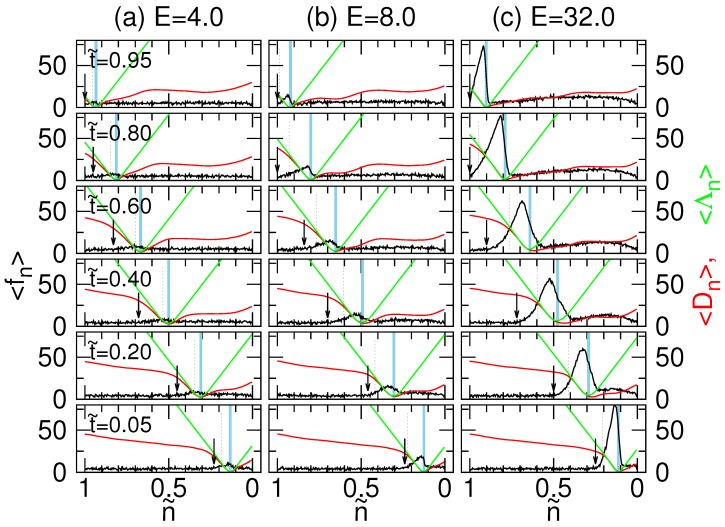
Variation of the bond tension $\langle f_{n}\rangle$ (the black curves) for N=256 at a set of scaled time points $\tilde{t}$ (values indicated in the figures) at the driving fields: (**a**) E=4.0; (**b**) 8.0; and (**c**) 32.0. The direction of the scaled $\tilde{n}$-axis is reversed so that the monomers entering the trans region stay on the right-hand side of the plot while the cis monomers rest on the left-hand side. The sky-blue region indicates the monomers in the pore region. The direct distance $\langle D_{n}\rangle$ and the contour distance $\langle \Lambda_{n}\rangle$ to the pore are plotted in red and green colors, respectively. The values of $\langle D_{n}\rangle$ and $\langle \Lambda_{n}\rangle$ are read from the right *y*-axis of the figure. In each plot, a downward arrow indicates the location of the tension front, whereas a dashed line marks the boundary for the straightened chain section.

To show the details, we plot the tension curves individually as a series of time points in Figure 6. We have reversed the direction of the $\tilde{n}$-axis in the plots and marked the pore region in a sky-blue color. Since the monomers with smaller $\tilde{n}$ arrive the right compartment (the trans region) earlier, the reversed plots present the propagation of tension compatible with the threading sense. It allows us to facilely understand the picture.

We can see that the tension $\langle f_{n}\rangle$ (the black curve) did exhibit a surge for E≥4.0, and the surge propagates from the chain head ($\tilde{n}=0)$ to the chain end ($\tilde{n}=1$) in a threading process. The surge is skewed with the left-hand part being broader. Both the width and the peak increase with *E*. The left border of the surge defines the tension front, which has been indicated in the plots by a downward arrow. To understand the phenomena further, we calculated two distances to the pore for each monomer *n*. The first distance $D_{n}$ is the direct distance to the pore, and the second one $\Lambda_{n}$ is the contour distance along the chain backbone to the pore. In each run, $D_{n}$ and $\Lambda_{n}$ are equal to zero if the number *n* denotes the monomer in the pore region. However, the mean values $\langle D_{n}\rangle$ and $\langle \Lambda_{n}\rangle$, averaged over the independent runs, are not. This is because at a given time, the monomers inside the pore may be different in different runs due to the stochastic nature of the threading process. Nonetheless, the minimum of $\langle D_{n}\rangle$ and $\langle \Lambda_{n}\rangle$ marks the most probable monomers in the pore at the moment. On the left of the minimum are the monomers in the cis region and on the right the ones in the trans region. $\langle \Lambda_{n}\rangle$ is approximately a linear increasing function away from the pore because the distance is proportional to the number of the monomers, which connect the monomer *n* to the pore. $\langle D_{n}\rangle$ displays a non-linear variation behavior on the two sides of the pore. On the trans side, the value is smaller, revealing that the sub-chain is closer to the wall than on the cis side. Noticeably, $\langle D_{n}\rangle$ acquires a value close to $\langle \Lambda_{n}\rangle$ in the cis region when *n* approaches the pore. The situation can happen only when the chain section is straightened by the driving force. The dashed lines in the figures depict the boundary of the straightened section, which are defined at $\langle D_{n}\rangle /\langle \Lambda_{n}\rangle =0.9$. We saw that the effect of straightening becomes more important when *E* gets stronger. When *E* is weak (refer to Figure S2 in the Supplementary Materials for E=0.2, 1.0 and 2.0), $\langle D_{n}\rangle$ is significantly smaller than $\langle \Lambda_{n}\rangle$. No straightening of the chain or surge of tension was observed.

The tension propagation theory [49,50,51,52] divides the driving forces into three regimes. In the weak force regime, the tension front propagates on the sub-chain, but the driving force is too weak to straighten any of the chain. In the intermediate force regime, the sub-chain is straightened near the pore entrance, but the tension front is farther than the boundary of the stretched chain section. In the strong regime, the force is so strong that the chain section within the tension front is completely straightened. Figure S2 shows that the thermal fluctuation can blur out the tension propagation in weak fields, and no tension front was seen. When E≥4.0 (Figure 6), we did observe the tension front propagating on the chain, but the boundary of the straightened section falls behind the front. Even at the extremely strong field E=32.0, the tension front remains larger than the straightened chain section. Therefore, the third force regime is not observed in our simulations.

### 4.3. Waiting Time

The waiting time is an important quantity to understand the dynamics of polymer translocation. It can be defined as the dwelling time for a threading chain being at a translocation state *s*. We calculated the waiting time function $w_{k}\left(s\right)$ for each simulation run *k*. Since *s* varies from one to *N*, the sum of $w_{k}\left(s\right)$ is equal to the translocation time:

(8) $$\tau_{k}=\sum_{s=1}^{N-1}w_{k}\left(s\right)=\int_{1}^{N-1}w_{k}\left(s\right)\;ds.$$

We remark that $w_{k}\left(s\right)$ is divergent at s=N because the chain dwells “permanently” at the last state when the threading process is completed. The mean waiting time was calculated by:

(9) $$w\left(s\right)=\frac{1}{M}\sum_{k=1}^{M}w_{k}\left(s\right)$$

over the *M* independent runs. In this study, M=500 for every set of the (N,E)-parameters. For comparison, the waiting time function was further normalized to be:

(10) $$\tilde{w}\left(\tilde{s}\right)=\frac{Nw(s)}{\langle \tau \rangle }.$$

**Figure 7:**
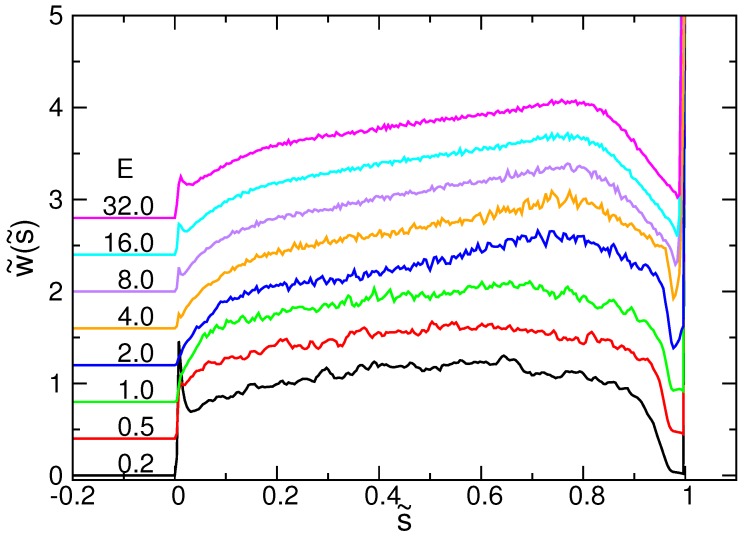
Normalized waiting time function $\tilde{w}\left(\tilde{s}\right)$ for N=256 at different field strengths *E* indicated at the left of the curves. $\tilde{s}$ is defined as s/N. The curves have been shifted upward with a fixed value, one after the other.

The normalized waiting time distribution satisfies the relation $\int_{0}^{1}\tilde{w}\left(\tilde{s}\right)\;d\tilde{s}=1$ for any values of *N* and *E*. Figure 7 shows the results for N=256 at different driving fields *E*. For clarity of the figure, the curves have been shifted upward, one after the other, by a fixed distance.

Near $\tilde{s}=0$, a sharp peak shows up suddenly on the curve. It is a responsive behavior to the switching-on of the electric field. The peak value is high at the weak field E=0.2, because considerable time is spent on overcoming the entropic force, which tends to drive the chain out of the pore in the reverse direction. When *E* increases, the peak value decreases significantly. The peak re-grows as E>4.0, showing that the inertia of the chain becomes important in the strong fields. During the main course of translocation, the waiting time function displays a hump. The hump is about mirror-symmetric to the middle point $\tilde{s}=0.5$ for E=0.2 and 0.5. It is the feature of a near-equilibrium process. The symmetry is broken when *E* increases, and the hump becomes more and more peaked, being distorted toward $\tilde{s}=0.8$. The asymmetry is the sign of far-from-equilibrium. It has been predicted that the position of the hump is related to the extent of the chain tension driven by a strong field [41,65]. Before reaching the hump peak, the tension front propagates on the chain in the cis region. It is the “tension propagation” stage. After the peak, the tension has arrived at the chain end, and the influence is exerted on the whole sub-chain. The dynamics enters the second stage, the “tail retraction”. Our results do support this picture. It can be seen in Figure 6 that the tension front touches the chain end at the moment about 80% of the translocation time, which corresponds to the state $\tilde{s}≃0.8$.

An overshoot occurs near $\tilde{s}=1$. Two effects cause the result. First, at the last moment of threading, the chain end enters the pore region, and thus, the number of monomers decreases in the pore. This results in a reduction of the driving force, which slows down the process and increases the waiting time. The second is the crowding effect. It derives from a fast translocation, in which the monomers entering the trans region have not enough time to be relaxed and are still crowded near the pore exit. Moreover, some monomers can be occasionally pushed back to the pore unless the main body of the chain diffuses into the semi-space. Therefore, the waiting time significantly increases for the last few *s* when E≥8.0.

**Figure 8:**
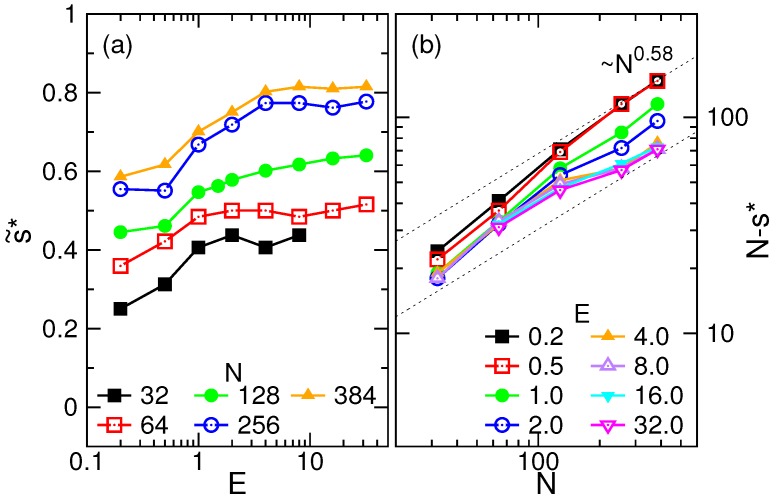
(**a**) Hump position ${\tilde{s}}^{*}$ of the normalized waiting time function $\tilde{w}\left(\tilde{s}\right)$, as a function of *E*. The number of monomers *N* is indicated in the figure. (**b**) $\left(N-s^{*}\right)$ vs. *N* at different field strengths *E*.

We went further to study the hump position ${\tilde{s}}^{*}$ of $\tilde{w}\left(\tilde{s}\right)$ in the main course of the translocation. The results are presented in Figure 8a as a function of *E*. At a given *N*, the position ${\tilde{s}}^{*}$ increases with *E* and saturates to some value. A longer chain implies a larger ${\tilde{s}}^{*}$. Since the hump position defines the transition from the tension-propagation stage to the tail-retraction one, the maximum extent of the tension on the chain is given by $N-s^{*}$. By plotting the range vs. *N* in Figure 8b in the log-log plot, we found that $N-s^{*}$ displays asymptotically a scaling behavior as $N^{0.58}$. It could be used to estimate the occurrence of the tail retraction in a threading process under the long-chain limit.

### 4.4. Drift-Diffusion Properties

Translocation is often understood in the framework of a drift-diffusion process [32]. The 1D drift-diffusion equation reads as:

(11) $$\frac{\partial \;p(s,t)}{\partial \;t}=-\frac{\partial }{\partial \;s}\left(v(s)p(s,t)\right)+\frac{\partial^{2}}{\partial \;s^{2}}\left(D(s)p(s,t)\right)$$

where p(s,t) is the probability density to find a threading chain at a state *s* (the translocation coordinate) at time *t*. D(s) and v(s) are the diffusion coefficient and the drift velocity, respectively, both of which are functions of *s*.

The drift velocity in this case describes the rate of state transition and can be calculated via the waiting time function by:

(12) $$v\left(s\right)=\frac{ds}{dt}≃\frac{\Delta s}{\Delta t}=\frac{1}{w(s)}.$$

**Figure 9:**
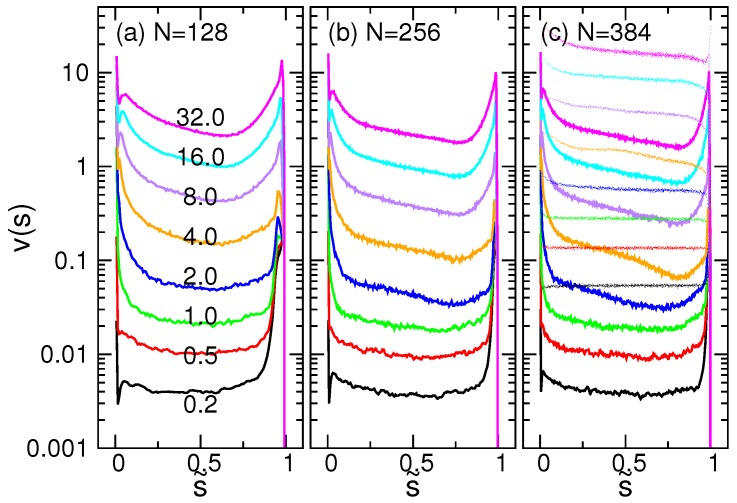
Drift velocity v(s) for: (**a**) N=128; (**b**) 256; and (**c**) 384. $\tilde{s}$ is the scaled translocation coordinate. The strength of the driving field *E* is indicated near the curve. In panel (c), the estimated drift velocity $v_{\mathrm{es}}\left(s\right)$ is plotted in thin dashed curves in the same color code.

The results for N=128, 256 and 384 are shown in Figure 9 at different driving fields *E*, plotted in the semi-log scale.

As expected, a larger *E* implies a larger v(s). At $\tilde{s}=0$, the value of v(s) is high. It decreases with increasing $\tilde{s}$ and reaches a minimum value at $\tilde{s}={\tilde{s}}^{*}$. When $\tilde{s}>0.8$, v(s) increases significantly, but drops sharply to zero as $\tilde{s}$ approaches the final stat, one. The magnitude and the profile of v(s) are similar to each other for different chain lengths.

Theorists have tried to understand the problem by giving the entropic free energy as:

(13) $$F_{\mathrm{entropic}}\left(s\right)=k_{B}T\left[\left(1-\gamma_{I}^{\prime }\right)\ln\left(N-s\right)+\left(1-\gamma_{\mathrm{III}}^{\prime }\right)\ln\left(s\right)\right],$$

where $\gamma_{I}^{\prime }$ and $\gamma_{\mathrm{III}}^{\prime }$ are the exponents whose values depend on the solvent conditions in the cis and trans regions, and the drift velocity was computed from the terminal speed of the monomers passing across the pore [66,67,68,69,72,88]. In this work, we chose $\gamma_{I}^{\prime }$ and $\gamma_{\mathrm{III}}^{\prime }$ to be 0.69 (the $\gamma^{\prime }$-value for self-avoiding chains [32,89]) and estimated the velocity by:

(14) $$v_{\mathrm{es}}\left(s\right)=\frac{f_{\mathrm{entropic}}+f_{\mathrm{driving}}}{\ell_{0}\zeta_{\mathrm{eff}}}.$$

The entropic force was obtained by taking the derivative of $F_{\mathrm{entropic}}\left(s\right)$ and is equal to:

(15) $$f_{\mathrm{entropic}}=\frac{k_{B}T}{N\ell_{0}}\left(1-\gamma^{\prime }\right)\frac{2\tilde{s}-1}{\tilde{s}\left(1-\tilde{s}\right)}.$$

The driving force was given by:

(16) $$f_{\mathrm{driving}}=eE\left(N_{m,\mathrm{II}}-N_{c,\mathrm{II}}^{\left(+1\right)}\right)$$

with $N_{m,\mathrm{II}}$ and $N_{c,\mathrm{II}}^{\left(+1\right)}$ being the numbers of the monomers and the condensed (+1)-ions in the pore region (II), respectively, and the effective friction coefficient was primarily calculated from the solvent friction, by:

(17) $$\zeta_{\mathrm{eff}}=\zeta \left(N_{m,\mathrm{II}}+N_{c,\mathrm{II}}^{\left(+1\right)}\right)$$

for the Rouse dynamics. The predicted $v_{\mathrm{es}}\left(s\right)$ have been plotted in Figure 9c in thin dashed curves, using the N=384 case for comparison. We saw that the profile of $v_{\mathrm{es}}\left(s\right)$ looks somewhat similar to v(s), but the value is about one order of magnitude larger. It suggests that the value of $\zeta_{\mathrm{eff}}$ was underestimated in the calculation. When a chain is driven into a narrow pore, the chain beads can have contact or get stuck momentarily with the wall beads. It significantly hinders the threading process, and thus, the effective friction should be much larger than the solvent friction. The other problem to be rectified is the divergence of the entropic force at the starting ($\tilde{s}=0$) and the ending ($\tilde{s}=1$) points of the process. The entropic free energy $F_{\mathrm{entropic}}\left(s\right)$, and hence, the force $f_{\mathrm{entropic}}$, were derived in the thermodynamic limit. It is valid only when the both sub-chain segments, *s* and (N−s), are large, which is just not the case near the process ends. Moreover, the non-equilibrium characteristics affect the system. For example, the inertia of threading and the crowding of the sub-chain should be considered, and the drift velocity should be more complicated than just the terminal speed.

The diffusion behavior in the translocation can be studied by calculating the variance of *s* as a function of time. The variance is defined by:

(18) $$\langle \Delta s^{2}\left(t\right)\rangle =\langle {\left(s\left(t\right)-\langle s\left(t\right)\rangle \right)}^{2}\rangle =\langle s^{2}\left(t\right)\rangle -{\langle s\left(t\right)\rangle }^{2}.$$

**Figure 10:**
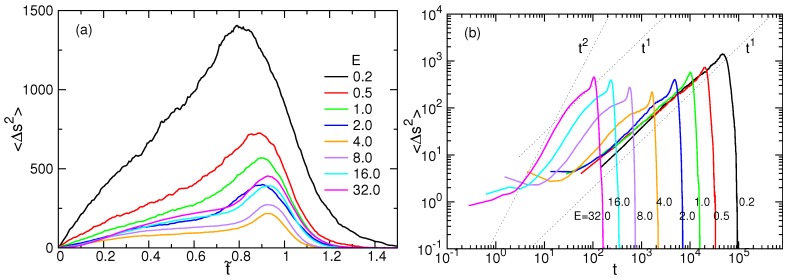
(**a**) Variance of the translocation coordinate $\langle \Delta s^{2}\rangle$ versus the scaled time $\tilde{t}$; (**b**) $\langle \Delta s^{2}\rangle$ plotted as a function of the real time *t* in the log-log plot. The chain has 256 monomers. The field strength *E* is indicated in the figures.

It measures the spreading of *s* at time *t*. Figure 10a presents the calculated results for N=256, plotted in the scaled time $\tilde{t}$.

$\langle \Delta s^{2}\rangle$ increases in the first part of the process and decreases to zero in the second part. It shows that the spreading of *s* is stopped at some moment and turns out to be a shrinkage. The reason for the change is that the chain must finish at the final state so that the variance is zero at the end. Therefore, the second part of the process must undergo a negative diffusion.

The logarithmic plots of $\langle \Delta s^{2}\rangle$ vs. *t* have been presented in Figure 10b. We found that in the weak fields E≤2.0, the variance scales approximately as $t^{\xi }$ and ξ=1 in the first part of the process. It is a normal diffusion behavior. As *E* increases, the scaling exponent *ξ* gradually augments and comes close to 2 at E=32.0, which describes a ballistic motion. The ballistic regime crosses over to a normal diffusion regime (ξ=1) with the evolution of time and then mounts to a super-diffusion one with ξ>2 due to the fast acceleration in the tail-retraction stage. The super-diffusion regime is small and soon switches to a negative diffusion to converge the variance to zero at the final part of the process.

**Figure 11:**
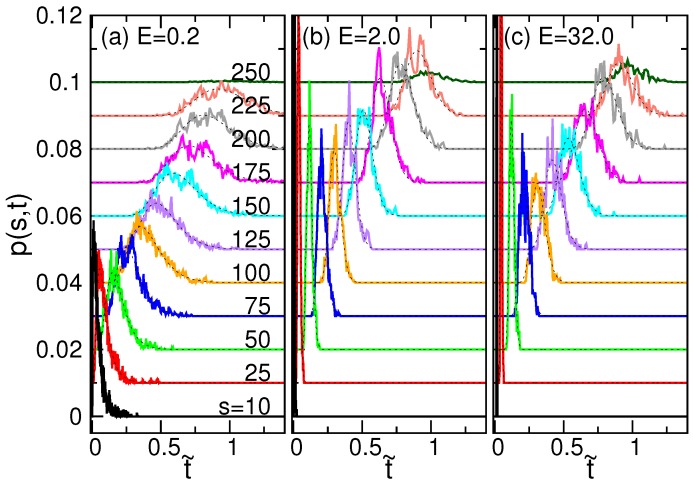
Probability densities p(s,t) versus the scaled time $\tilde{t}$ for (**a**) E=0.2, (**b**) 2.0 and (**c**) 32.0 at different translocation coordinates *s* indicated near the curves. For clarity, the curves have been shifted upward, one by one, by a fixed step. The dashed curves superimposed on the data are the results of fitting from Equation (19). The number of monomers of the chain *N* is 256.

The probability density distribution p(s,t) is directly studied from the simulations. Figure 11 shows p(s,t) at a weak, an intermediate and a strong driving field. The curves are plotted versus scaled time $\tilde{t}$ at different state values *s*. For clarity of the plots, the curves have been shifted upward by a fixed step, one after the other.

p(s,t) is a peaked function for a given *s*, and the peak moves to the right as *s* increases. In the semi-log plot, the curves look similar to a Gaussian distribution. Thus, we can fit the curves using a log-normal distribution under the form of:

(19) $$p\left(s,t\right)=\frac{A_{s}}{\sqrt{2\pi }\sigma_{s}t}\exp\left(-\frac{{\left(\ln t-\ln\mu_{s}\right)}^{2}}{2\sigma_{s}^{2}}\right)$$

where $A_{s}$, $\sigma_{s}$ and $\mu_{s}$ are three parameters, which depend on *s*. The normalization condition for p(s,t) is $\int_{1}^{N-1}\int_{0}^{\infty }p\left(s,t\right)\;dt\;ds=1$. Integrating the time integral yields $\int_{1}^{N-1}A_{s}\;ds=1$ where $A_{s}$ can be linked to the waiting time function by $A_{s}=w\left(s\right)/\langle \tau \rangle$. Therefore, we performed a two-parameter fit for the data of p(s,t) at each *s*, via $\sigma_{s}$ and $\mu_{s}$, using the information of waiting time for $A_{s}$. The fitting was done by the Levenberg–Marquardt method [90], and the resulting curves have been superimposed on the simulation data with dashed lines in Figure 11. Good matches of the fitting were observed.

**Figure 12:**
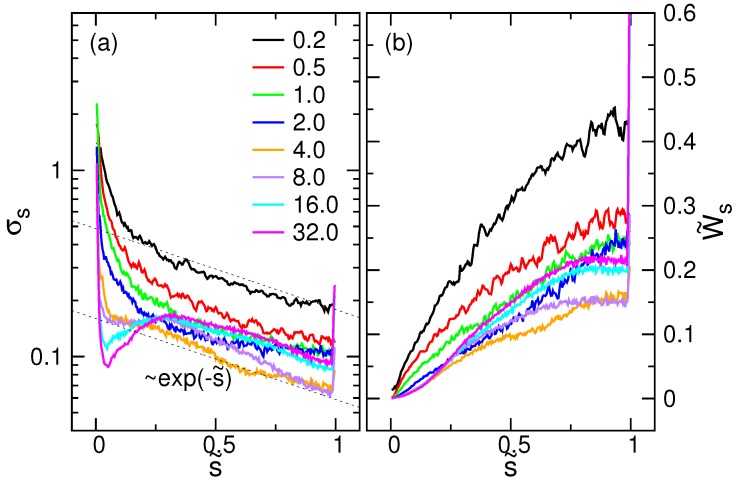
(**a**) Fitting parameter $\sigma_{s}$ as a function of the scaled coordinate $\tilde{s}$ at different *E* fields; (**b**) relative width ${\tilde{W}}_{s}$ of the log-normal distribution, calculated by $2\sinh\left(\sqrt{2\ln2}\sigma_{s}\right)\exp\left(-\sigma_{s}^{2}\right)\mu_{s}/\langle \tau \rangle$, versus $\tilde{s}$. The field strengths *E* are indicated in the figure. The number of monomers *N* is equal to 256.

The results for the fitting parameter $\sigma_{s}$ are presented in Figure 12a, as a function of $\tilde{s}$. When E<8.0, $\sigma_{s}$ decreases with *s*. If the field is strong, it drops sharply and then displays a peak at $\tilde{s}≃0.25$. Near $\tilde{s}=1$, the crowding effect due to fast translocation hinders the threading process and results in an overshoot. It is worth noticing that the $\sigma_{s}$ curves decreases approximately as exp(−s/N) for s>0.25N.

The “full width at half maximum” $W_{s}$ of the log-normal distribution is given by:

(20) $$W_{s}=2\sinh\left(\sqrt{2\ln2}\sigma_{s}\right)\exp\left(-\sigma_{s}^{2}\right)\mu_{s}$$

(see the notes on the log-normal distribution in the Supplementary Materials). Figure 12b presents the relative width ${\tilde{W}}_{s}=W_{s}/\langle \tau \rangle$ calculated from the two fitting parameters, $\sigma_{s}$ and $\mu_{s}$. At a given *E*, the width is an increasing function of *s* and tends to saturate to a value as $\tilde{s}$ approaches one. The whole curve ${\tilde{W}}_{s}$ decreases (moves downward) firstly with *E*, up to E=4.0, and then turns to increase (moves upward) with the field strength.

The mean time needed to reach a translocation state *s* is calculated by:

(21) $$\langle t_{s}\rangle =\int_{0}^{\infty }tp\left(s,t\right)\;dt.$$

**Figure 13:**
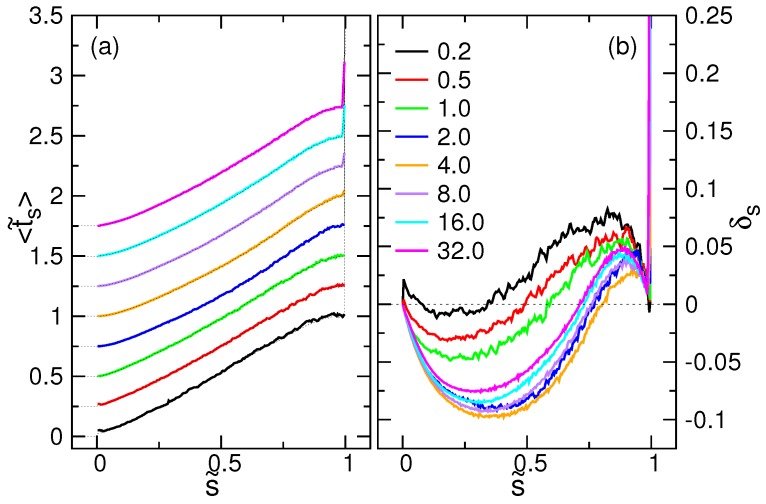
(**a**) $\langle {\tilde{t}}_{s}\rangle$ calculated by $\mu_{s}\exp\left(\sigma_{s}^{2}/2\right)$, plotted in the scaled coordinate $\tilde{s}$ at different *E*. For clarity, the curves have been shifted upward, one by one, by a fixed value. The strengths of *E* are indicated in the figure. The chain has 256 monomers. The dashed curves are the mean time obtained directly from the simulations, superimposed here for comparison. (**b**) Retardation $\delta_{s}$ as a function of the scaled coordinate $\tilde{s}$.

It can be calculated from the two fitting parameters by $\langle t_{s}\rangle =\exp\left(\sigma_{s}^{2}/2\right)\mu_{s}$. The scaled mean time function $\langle {\tilde{t}}_{s}\rangle =\langle t_{s}\rangle /\langle \tau \rangle$ is plotted in Figure 13a for different *E* fields. The results are consistent with the mean time directly obtained from the simulations, $\frac{1}{M}\sum_{k=1}^{M}t_{s,k}$, which have been superimposed on the data by dashed lines. The dashed curve is the inverse mapping of the number of monomers $\langle N_{m,\mathrm{III}}\rangle$ versus $\tilde{t}$ given in Figure 7 of the paper [44].

To understand the dynamics more in detail, we calculated the retardation:

(22) $$\delta_{s}=\frac{1}{\langle \tau \rangle }\left(\langle t_{s}\rangle -\frac{s}{\bar{v}}\right)$$

which measures the relative time delay of $\langle t_{s}\rangle$ to the “scheduled” time, $s/\bar{v}$, estimated from the mean threading speed $\bar{v}=N/\langle \tau \rangle$. The results are presented in Figure 13b.

The $\delta_{s}$ curve looks like a lying S. In the first stage of translocation, the threading is ahead of the schedule because $\delta_{s}<0$. It becomes behind the schedule ($\delta_{s}>0$) in the second stage of the process and finally catches up to the “plan” when $\tilde{s}$ approaches one. The overshoot at $\tilde{s}=1$ reflects the fact that the chain stays definitely at the last state, and thus, $\langle t_{s}\rangle$ diverges as seen in Figure 13a. The zero of $\delta_{s}$ denotes the intersection of the $t_{s}$ curve with the scheduled time. Since the threading speed can be calculated by $v\left(s\right)=d\langle s\rangle /dt={\left(d\langle t_{s}\rangle /ds\right)}^{-1}$, the intersection is near the inflection point of the $\langle t_{s}\rangle$, which is, in fact, at $\tilde{s}={\tilde{s}}^{*}$, the smallest threading speed that occurs. The minimum and the maximum of $\delta_{s}$ are located by $d\delta_{s}/ds=0$. At the two extreme points, the threading speed is exactly $\bar{v}$.

## 5. Conclusions

We have investigated the conformation changes of the sub-chains on the two sides of the separating membrane wall during a translocation. The calculations of the shape factor *η*, the asphericity 〈A〉 and the prolateness 〈P〉 showed that the cis-side sub-chain was elongated with passing time even in a weak driving field. The stronger the driving field, the larger the degree of elongation. The trans-side sub-chain, on the other hand, was compressed. It became an oblate object in strong fields. The study of the orientation revealed that the sub-chain lay down in the trans region and formed a disk-like object in contact with the wall under a fast translocation condition. The phenomena reflected the important cis-trans dynamical asymmetry where the sub-chains were subjected to a pulling and a pushing force in the two regions, respectively.

The study of the bond stretching showed that a surge of tension was displayed on the chain when the driving force was strong. In weak fields, the surge was smeared out by the thermal fluctuation. We have located the tension front and calculated the averaged direct distance $\langle D_{n}\rangle$ and the contour length $\langle \Lambda_{n}\rangle$ to the pore for each monomer. From the ratio $\langle D_{n}\rangle /\langle \Lambda_{n}\rangle$, we were able to determine the chain section, which was straightened in the cis region. The tension front was found farther than the section boundary. The predicted force regime with the section boundary extending up to the front in the tension propagation theory was not observed. Furthermore, with the help of the tension calculation, the hump appearing on the main body of the waiting time function was shown to be the turning point from the tension-propagation stage to the tail-retraction stage in the translocation.

The drift velocity of translocation v(s) was calculated and compared with the theoretical prediction. A large discrepancy was found, which could be attributed to the underestimation of the effective friction coefficient. Furthermore, the divergence of the entropic force at the two ends of the process revealed that the free energy of the system should be further revised. The variance of the translocation coordinate, $\langle \Delta s^{2}\rangle$, was studied as a function of time. The results showed that the translocation must be ended with a negative diffusion behavior. In weak fields, the translocation started with a normal diffusion. A ballistic behavior showed up if the field became strong, and the process crossed over to a normal diffusion and then super-diffusion before entering the last, negative diffusion stage. Finally, the probability density distribution p(s,t) was fitted by a log-normal distribution in the *t* space for each *s*. The width $W_{s}$ of the distribution was an increasing function of *s* and saturated to some value when *s* approaches the final state *N*. The mean time $\langle t_{s}\rangle$ to reach a state *s* was computed. Through the calculation of the retardation $\delta_{s}$, we showed that the state of translocation went ahead of the schedule estimated from the averaged transition rate in the tension-propagation stage, while in the tail-retraction stage, it was behind schedule until the end of the process. Our study has highlighted many detailed physics and dynamics of polyelectrolytes threading through a pore and has verified several theoretical aspects. It provides valuable information for researchers to further understand and solve the problem. The obtained knowledge could also be extended to understand the diffusion and assemblies of polyelectrolytes in nanoporous materials for a wide range of applications [91,92].

## Supplementary Materials

The followings are available online at http://www.mdpi.com/2073-4360/8/10/378/s1. 1. Mapping translocation time to real time. 2. Derivation of the shape factors for a spherical and a disk-like object. 3. Azimuthal angle of the principal axis of the sub-chains in the cis and trans regions. 4. Variation of the bond tension at weak driving fields. 5. Notes on the log-normal distributions.

## List of Symbols:

〈X〉	Mean value of physical quantity *X*
$X_{I}$, $X_{\mathrm{II}}$, $X_{\mathrm{III}}$	Values of physical quantity *X* in the cis (I), pore (II) and trans (III) regions
*A*	Asphericity
*α*, *δ*	Exponents for the scaling behavior of $\langle \tau \rangle ∼N^{\alpha }E^{-\delta }$
$\delta_{s}$	Retardation
$C_{s}$	Salt concentration
$D_{n}$	Direct distance from the *n*-th monomer to the pore
D(s)	Diffusion coefficient
*E*	Strength of electric field (applied only inside the pore)
*e*	Charge unit
($\varepsilon_{\mathrm{bb}}$, $\sigma_{\mathrm{bb}}$), ($\varepsilon_{\mathrm{bw}}$, $\sigma_{\mathrm{bw}}$)	Energy and length parameters of WCA potential for bead-bead (bb) and bead-wall (bw) interactions
$f_{n}$	tensile force on the *n*-th bond
*I*	Ionic strength
$\left[G\right]$	Gyration tensor
*κ*	Inverse Debye length
*k*	Spring constant of bond
$k_{B}$	Boltzmann constant
$\ell_{0}$	Equilibrium bond length
$\ell_{c}$	Charge distance on the chain backbone
$\ell_{n}$	Length of the *n*-th bond
$\ell_{p}$, $\ell_{p,0}$, $\ell_{p,e}$	Persistence length, intrinsic and electrostatic persistence lengths
*η*	Shape factor ($=\langle R_{e}^{2}\rangle /\langle R_{g}^{2}\rangle$)
$\Lambda_{n}$	Contour distance from the *n*-th monomer to the pore
$\lambda_{B}$	Bjerrum length
$\lambda_{1}$, $\lambda_{2}$, $\lambda_{3}$	Eigenvalues of the gyration tensor $\left[G\right]$
$\bar{\lambda }$	Mean of the eigenvalues ($\bar{\lambda }=\left(\lambda_{1}+\lambda_{2}+\lambda_{3}\right)/3$)
*m*	Mass unit of simulation
*N*	Number of monomers of a chain
$N_{c}^{\left(+1\right)}$, $N_{c}^{\left(-1\right)}$	Number of condensed (+1)-ions, number of condensed (−1)-ions
$N_{m}$	Number of monomers in a region
*n*, $\tilde{n}$	Monomer ID number, scaled *n* ($\tilde{n}=n/N$)
*ϕ*	Azimuthal angle
*P*	Prolateness
p(s,t)	Probability density distribution
$R_{e}^{2}$	Square of the end-to-end distance
$R_{g}^{2}$	Square of the radius of gyration
*σ*	Length unit of simulation
$\sigma_{s}$, $\mu_{s}$	Fitting parameters for Equation (19)
*s*, $\tilde{s}$	Translocation coordinate, scaled *s* ($\tilde{s}=s/N$)
$s^{*}$, ${\tilde{s}}^{*}$	Hump position of the w(s) function, scaled $s^{*}$ (${\tilde{s}}^{*}=s^{*}/N$)
*θ*	Polar angle
*T*	Temperature
*t*, $\tilde{t}$	Time, scaled time ($\tilde{t}=t/\langle \tau \rangle$)
$t_{s}$, ${\tilde{t}}_{s}$	Time needed to reach the translocation coordinate *s*, ${\tilde{t}}_{s}=t_{s}/\langle \tau \rangle$
*τ*	Translocation time
$\tau_{u}$	Time unit of simulation
v(s), $v_{\mathrm{es}}\left(s\right)$	Drift velocity, estimated drift velocity
$\bar{v}$	Mean threading velocity ($\bar{v}=N/\langle \tau \rangle$)
$W_{s}$, ${\tilde{W}}_{s}$	Full width at half maximum of a log-normal distribution, ${\tilde{W}}_{s}=W_{s}/\langle \tau \rangle$
w(s), $\tilde{w}\left(\tilde{s}\right)$	Waiting time function, normalized waiting time function
*ξ*	Exponent for the scaling behavior of $\langle \Delta s^{2}\rangle ∼t^{\xi }$
*ζ*	Friction coefficient
