# Supplementary material for: Conformation Change, Tension Propagation and Drift-Diffusion Properties of Polyelectrolyte in Nanopore Translocation
Source: Polymers (Basel). 2016 Oct 24;8(10):378. doi: 10.3390/polym8100378 (PMC6432159; doi:10.3390/polym8100378)
Supplement: Supplementary file 1 [file polymers-08-00378-s001.pdf]

Supplementary Materials: Conformation Change, Tension Propagation and Drift-Diffusion Properties of Polyelectrolyte in Nanopore Translocation

Pai-Yi Hsiao

1. Mapping Translocation Time to Real Time

In this study, we choose:

$$\begin{aligned} \sigma &= 2.38 \times 10^{-10}\,\mathrm{m} \\ m &= 200\,\mathrm{g/mol} = 3.32 \times 10^{-25}\,\mathrm{kg} \\ \tau_\mathrm{u} &= 2.13 \times 10^{-12}\,\mathrm{s} \\ e &= 1.602 \times 10^{-19}\,\mathrm{C} \end{aligned}$$

as the length, mass, time and charge units of our simulation system, respectively. The mean translocation time is investigated under different conditions [44]. For example, at the weak driving field $E = 0.2\,k_\mathrm{B}T/(e\sigma)$, the mean translocation time is $\langle\tau\rangle = 88757.9\,\tau_\mathrm{u}$ for $N = 384$. It yields an average threading time $231.1\,\tau_\mathrm{u}$ per monomer, which corresponds to 0.492 ns in the real time unit. This average threading time is about one to two orders of magnitude shorter than a typical threading time, 5 to 30 ns per base pair, observed in DNA translocation experiments [58]. The reason for this discrepancy can be attributed to the setting of the monomer friction coefficient ζ to a small value of $1.0\,m\tau_\mathrm{u}^{-1}$ in the Langevin dynamics simulations (refer to Equation (4) in the paper [44]). In an aqueous solution, the friction coefficient for a base pair can be estimated by Stokes' law $\zeta_\mathrm{w} = 3\pi\mu d$, which gives a value of $8.48 \times 10^{-12}\,\mathrm{kg}\cdot\mathrm{s}^{-1}$ if we take the water viscosity $\mu = 9 \times 10^{-4}\,\mathrm{Pa}\cdot\mathrm{s}$ and set the monomer diameter to $d \simeq 1\,\mathrm{nm}$. Therefore, an appropriate value for ζ should be $\zeta_\mathrm{w} = 54.4\,m\tau_\mathrm{u}^{-1}$, which is about 50-times larger than the current value. The small value of ζ was used voluntarily, for the purpose of increasing the particle moving speed, which reduces the needed simulation steps and, thus, accelerates the threading process. Therefore, the results related to time in the simulations should be primarily corrected by multiplying the factor 54.4. The obtained average threading time per monomer is hence corrected to be 26.8 ns, which agrees well with the experiments.

2. Deriving the Value of the Shape Factor

2.1. *η for an Ideal Chain Forming a Sphere*

Let R be the radius of the sphere. Assume that the chain ends are randomly distributed inside the sphere and that the probability density to find a monomer is a constant, $P(\vec{r}) = 1/(\frac{4}{3}\pi R^3)$. We can calculate the mean square of the radius of gyration from the formula:

$$\langle R_\mathrm{g}^2\rangle = \int r^2 P(\vec{r})\, d^3\vec{r} = \int_0^R \int_0^{2\pi} \int_0^{\pi} \frac{r^4 \sin\theta\, d\theta\, d\phi\, dr}{\frac{4}{3}\pi R^3} = \frac{3}{5}R^2 \qquad (1)$$

where the triple integral has been performed in the spherical coordinates (r, θ, ϕ). The mean square of the end-to-end distance can be calculated by:

$$\langle R_\mathrm{e}^2\rangle = \int\int |\vec{r}_1 - \vec{r}_N|^2 P(\vec{r}_1) P(\vec{r}_N)\, d^3\vec{r}_1\, d^3\vec{r}_N \qquad (2)$$

Apply the law of cosines $|\vec{r}_1 - \vec{r}_N|^2 = r_1^2 + r_N^2 - 2r_1 r_N \cos\theta_{1N}$, where θ_{1N} is the angle between vectors $\vec{r}_1$ and $\vec{r}_N$. Change the variable θ_1 to the variable θ_{1N} and perform the integration for the variables θ_N, ϕ_N and ϕ_1. We obtain:

$$\langle R_e^2 \rangle = \frac{2\pi \cdot 4\pi}{(\frac{4}{3}R^3)^2} \int_0^R \int_0^R \int_0^\pi (r_1^2 + r_N^2 - 2r_1 r_N \cos\theta_{1N}) r_1^2 r_N^2 \sin\theta_{1N}\, d\theta_{1N}\, dr_1\, dr_N = \frac{6}{5}R^2 \tag{3}$$

The shape factor $\eta \equiv \langle R_e^2 \rangle / \langle R_g^2 \rangle$ can be then computed and is equal to two.

2.2. *η for an Ideal Chain Forming a Disk*

Let R be the disk radius. Assume the chain ends are randomly distributed inside the disk and that the probability density to find a monomer is $P(\vec{r}) = 1/(\pi R^2)$. Similar to the derivation in Section 2.1, the integral can be done in the polar coordinates (r, ϕ). We obtain:

$$\langle R_g^2 \rangle = \int r^2 P(\vec{r})\, d^2\vec{r} = \int_0^R \int_0^{2\pi} \frac{r^3\, d\phi\, dr}{\pi R^2} = \frac{R^2}{2} \tag{4}$$

$$\langle R_e^2 \rangle = \int\int |\vec{r}_1 - \vec{r}_N|^2 P(\vec{r}_1) P(\vec{r}_N)\, d^2\vec{r}_1\, d^2\vec{r}_N = R^2 \tag{5}$$

which yields $\eta = 2$, as well.

3. Azimuthal Angle of the Principal Axis of the Sub-chains

The averaged azimuthal angle $\langle\phi\rangle$ of the principal axis of the sub-chains in the cis region (I) and trans region (III) are plotted in Figure S1. Owing to the symmetry in the transverse direction, the azimuthal curve fluctuates around 0°.

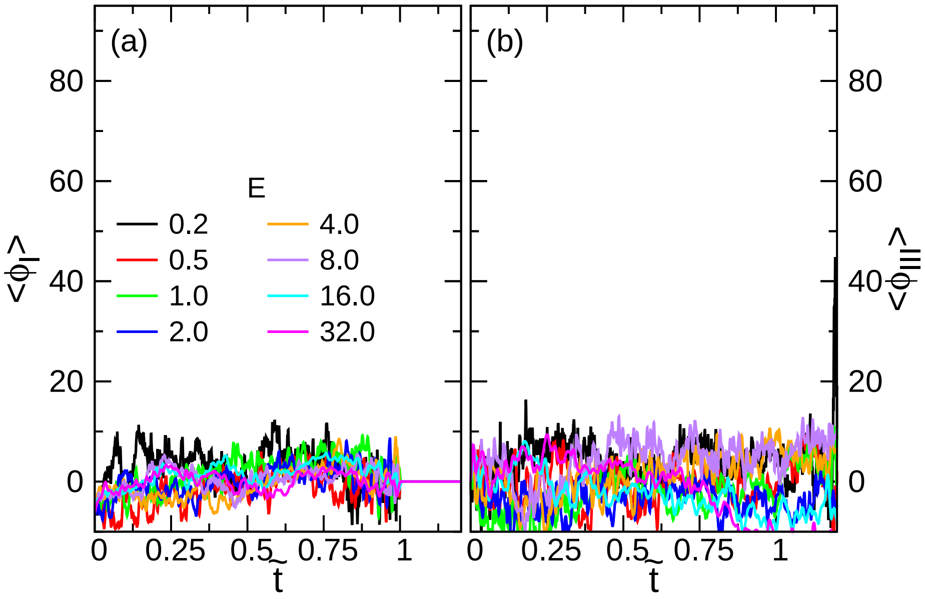

Figure S1. Variations of the averaged azimuthal angle, (**a**) $\langle\phi_I\rangle$ and (**b**) $\langle\phi_{III}\rangle$ (in degree °), as a function of the scaled time $\tilde{t}$ at different driving electric fields E. The chain has 256 monomers.

4. Variation of the Tension Force at Weak Driving Fields

The tension force $\langle f_n \rangle$ for each bond n at $E = 0.2$, 1.0 and 2.0 $k_B T/(e\sigma)$ were calculated and presented in Figure S2. For analysis, the averaged direct distance $\langle D_n \rangle$ and the averaged contour distance $\langle \Lambda_n \rangle$ for each monomer n to the pore were also plotted in the same figure. We can see that the

thermal fluctuation blurs out the surge of tension, and therefore, no tension front is observed. $\langle \Lambda_n \rangle$ is significantly larger than $\langle D_n \rangle$ so that the sub-chain is not straightened near the pore entrance.

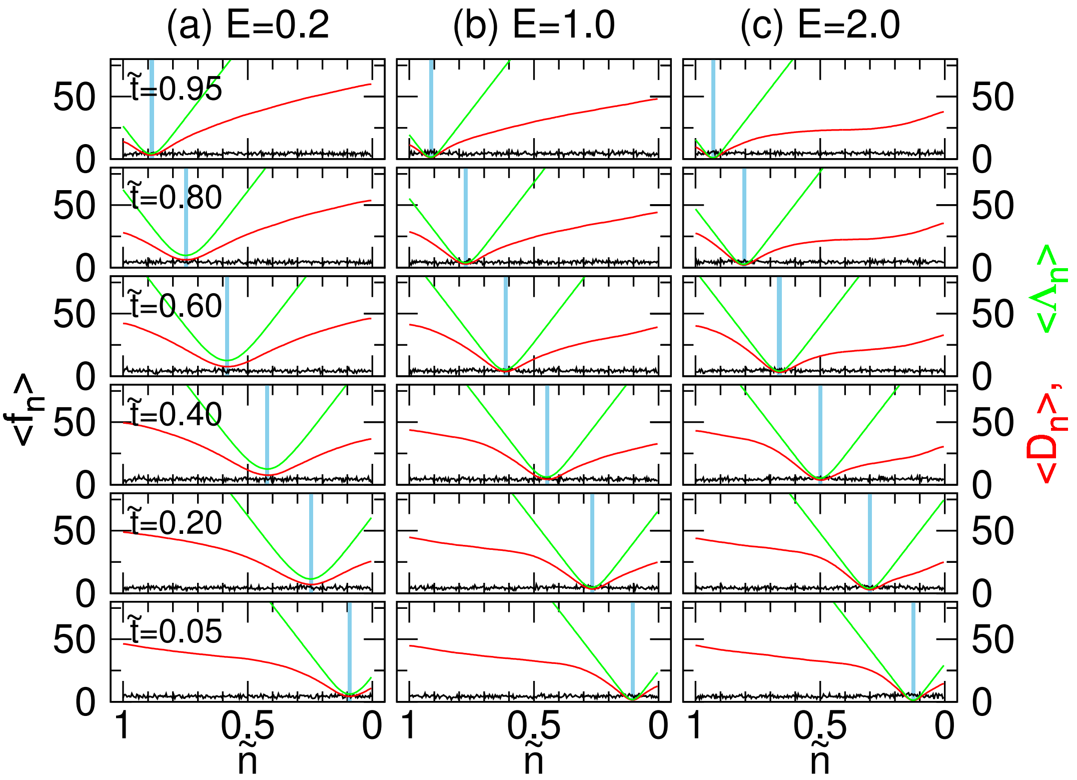

Figure S2. Variation of bond tension $\langle f_n \rangle$ (the black curves) for $N = 256$ at a set of scaled time points (values indicated in the figures) at the weak driving fields: (**a**) $E = 0.2$; (**b**) 1.0; and (**c**) 2.0. The direction of the $\tilde{n}$-axis is reversed so that the monomers entering the trans-region stay on the right-hand side of the plot, while the cis monomers rest on the left-hand side. The sky-blue region indicates the monomers in the pore region. The direct distance $\langle D_n \rangle$ and the contour distance $\langle \Lambda_n \rangle$ to the pore are plotted in red and green colors, respectively. The values of $\langle D_n \rangle$ and $\langle \Lambda_n \rangle$ are read from the right y-axis in the figure.

5. Notes on the Log-Normal Distribution

Given two parameters μ and σ, the log-normal distribution is defined by:

$$p(x) = \frac{1}{\sqrt{2\pi}\sigma x} \exp\left(-\frac{(\ln x - \ln \mu)^2}{2\sigma^2}\right). \tag{6}$$

It is the probability density function of a random variable X, whose logarithm satisfies the Gaussian (normal) distribution with the mean μ and the standard deviation σ. The maximum (or called "the mode") of the log-normal distribution occurs at $x = x_{\max} \equiv \mu \exp(-\sigma^2)$, and the value is:

$$p(x_{\max}) = \frac{1}{\sqrt{2\pi}\sigma\mu} \exp\left(\frac{\sigma^2}{2}\right). \tag{7}$$

The median of the distribution occurs at $x = \mu$ because $\int_0^\mu p(x)\,dx = 0.5$, and the mean is located at $x = \langle x \rangle = \mu \exp(\sigma^2/2)$. In general, the n-th moment of the log-normal variable X is given by:

$$\langle x^n \rangle \equiv \int_0^\infty x^n p(x)\,dx = \mu^n \exp\left(\frac{n^2\sigma^2}{2}\right) \tag{8}$$

and thus, the variance is equal to:

$$\langle \Delta x^2 \rangle = \langle x^2 \rangle - \langle x \rangle^2 = \mu^2 e^{\sigma^2} \left(e^{\sigma^2} - 1 \right). \tag{9}$$

The full width at half maximum (FWHM) defines the width of a distribution function measured between two points surrounding the maximum at which the distribution has a value of half of the maximum value. For example, the half maximums of a Gaussian distribution $\frac{1}{\sqrt{2\pi}\sigma} \exp\left(-\frac{(x-\mu)^2}{2\sigma^2}\right)$ occur at $x_{1,2} = \mu \pm \sqrt{2\ln 2}\sigma$, and therefore, the FWHM is $x_1 - x_2 = 2\sqrt{2\ln 2}\sigma$. For the log-normal distribution discussed here, the half maximums occur at the two points $x_1 = e^{\sqrt{2\ln 2}\sigma} x_{\max}$ and $x_2 = e^{-\sqrt{2\ln 2}\sigma} x_{\max}$. Therefore, the FWHM is $2\sinh(\sqrt{2\ln 2}\sigma) x_{\max}$.
